# Casting and Characterization of A319 Aluminum Alloy Reinforced with Graphene Using Hybrid Semi-Solid Stirring and Ultrasonic Processing

**DOI:** 10.3390/ma15207232

**Published:** 2022-10-17

**Authors:** Bernoulli Andilab, Payam Emadi, Comondore Ravindran

**Affiliations:** Centre for Near-Net-Shape Processing of Materials, Toronto Metropolitan University, Toronto, ON M5B 2K3, Canada

**Keywords:** aluminum alloys, metal matrix composites, graphene, mechanical properties, metalcasting, strengthening mechanisms

## Abstract

Advanced metallurgical processing techniques are required to produce aluminum matrix composites due to the tendency of the reinforcement particles to agglomerate. In this study, graphene nano-platelet reinforcement particles were effectively incorporated into an automotive A319 aluminum alloy matrix using a liquid metallurgical route. Due to its low density, it is a highly difficult task to produce an aluminum matrix composite reinforced with graphene. Hence, this study explored a novel approach to prevent particle floating to the melt surface and agglomeration. This was achieved via a hybrid semi-solid stirring of A319, followed by ultrasonic treatment of the liquid melt using a sonication probe. The microstructure and graphene particles were characterized using optical microscopy and scanning electron microscopy. Furthermore, the interfacial products produced with the incorporation of graphene in liquid aluminum were analyzed with X-ray diffraction. The tensile test results exhibited 10, 11 and 32% improvements in ultimate tensile strength, yield strength, and ductility of A319 reinforced with 0.05 wt.% addition of graphene. Analysis of strengthening models demonstrated primary contribution from Hall-Petch followed by CTE mismatch and load bearing mechanism. The results from this research enable the potential for using cost-effective, efficient and simple liquid metallurgy methods to produce aluminum reinforced graphene composites with improved mechanical properties.

## 1. Introduction

High-strength aluminum (Al) alloys, such as the A319 alloy, are commonly used in the production of lightweight and high-performance automotive powertrain components. Continuous efforts are made by researchers to improve the properties of Al alloys including through the use of modification, grain refinement and heat treatment. An alternative strengthening method is through the reinforcement of metals with hard ceramic particles has gained impetus to produce metal matrix composites (MMCs). MMCs are manufactured by dispersing the reinforcements in the metal matrix. The reinforcements are typically applied to improve the properties of the base metal such as strength, ductility, and thermal conductivity. Aluminum and its alloys have attracted much attention as a base metal in MMCs [1,2]. Aluminum metal matrix composites (AMCs) are widely used as structural components in aircraft, aerospace, automobiles and various other fields [3]. The most commonly used reinforcements are silicon carbide (SiC), aluminum oxide (Al_2_O_3_) and titanium boride (TiB_2_). These reinforcements have been found to increase the tensile strength, hardness, density and wear resistance of Al and its alloys [3].

In recent years, graphene has emerged as a material with high potential as a reinforcement particle for AMCs. Graphene is an atomic-scale honeycomb lattice made of carbon atoms. It has unique properties such as high strength and thermal conductivity [4,5]. Graphene is comparatively a newer type of carbon-based nanoparticles. Graphene can be effectively used as reinforcement for structural composites due to its unique characteristics (2-D single-atom-sheet sp^2^ hybridized carbon atoms) and significantly enhanced mechanical and thermal properties [6].

The particle distribution plays a very vital role in the properties of the AMCs, which is improved by intensive shearing of the liquid melt or rigorous mixing of metallic powders. As a result, appropriate processing of AMCs reinforced with graphene is key in developing a material with enhanced properties. Optimized dispersion of graphene reinforcement in the Al matrix plays a critical role in improving the properties of AMCs. This is difficult to achieve because of the strong van der Waals forces between graphene particles, high specific surface area (2630 m^2^/g) and low density of graphene (~1.8–2.2 g/cm^3^) compared to Al (~2.7 g/cm^3^). As a result, various processing techniques have been explored to determine the optimal method for incorporating graphene into Al.

The most commonly used processing route for the production of graphene reinforced AMCs is powder metallurgy [7,8,9]. However, it should be noted that powder metallurgy processes are typically lengthy and involve several steps, including final processing such as rolling or extrusion. For example, Bhaudaria et al. [3] investigated the combined effect of a nanocrystalline matrix and reinforcement with 0.5 wt.% graphene nano-platelets (GNPs) on pure Al using ball milling and spark plasma sintering. It was found that the ultimate tensile strength improved by 85% compared to that of pure Al. However, the increase in strength was accompanied by a significant decrease in ductility [3]. The decrease in ductility was attributed to cracking at the Al-graphene interface. It is also well known that agglomeration is an issue during processing of AMCs reinforced with graphene, which leads to porosity and a reduction in mechanical properties [10,11]. The results demonstrate excellent potential due to the significant strengthening enhancement, however the process involves multiple methods which can increase time and cost. Therefore, while powder metallurgy can enable the production of AMCs with enhanced mechanical properties, it may not be practical for industrial applications due to time and cost constraints.

As an alternative, liquid metallurgy techniques such as casting have been explored as a processing route to produce graphene reinforced AMCs. However, stir casting is not typically used compared to powder metallurgy techniques when manufacturing AMCs reinforced with graphene. This is due to the density difference between graphene and Al, which results in the floating of graphene particles at or near the melt surface. This leads to severe losses of graphene particles during melt treatment and casting. Further, the poor wettability between graphene and Al can inhibit the interfacial interaction which leads to agglomeration, thereby deteriorating the mechanical properties of the composite. Nonetheless, some studies have shown success in reinforcing Al with graphene. Liquid metallurgy processing techniques such as casting are of interest to the industry because they are practical, while enabling mass production capability. This is especially important for the automotive industry, where powder metallurgy techniques are less feasible.

Alipour and Eslami-Farsani [12] reported an optimal increase in tensile strength from 263 to 372 MPa after 0.5 wt.% addition of GNPs in an AA7068 Al alloy through a stir casting process. The main mechanism for this improvement in tensile strength was due to the grain refinement, according to the authors. The authors also found that beyond 0.5 wt.% addition, severe agglomeration of GNPs occurred. Similarly, Srivistava et al. [13] used the stir casting method to reinforce AA1100 Al alloy with GNPs. The results showed an increase in tensile strength and yield strength with increasing addition of GNPs up to 1.2%. In contrast, the elongation was negatively affected resulting in a brittle failure mode. Of note, while these studies implemented the stir casting technique, preprocessing via ball milling was required to prepare Al-graphene master alloys in order to incorporate graphene into the liquid Al melt. In the current study, the authors present a method which enables the incorporation of graphene without the requirement of preprocessing.

An alternative liquid metallurgy technique that has been successfully implemented to reinforce Al with graphene is via the pressure infiltration method [14,15]. It was found that the tensile strength of an Al-20Si alloy can be improved by 230% using the pressure infiltration method to reinforce 1.5 wt.% of graphene into the matrix [14]. This is also an effective method, however certain components such as automotive engine blocks and cylinder heads are not produced using pressure infiltration due to their complex geometry. Hence, there is a need for further exploration of methods that enable the production of high-strength AMCs reinforced with graphene.

Conventional casting and pressure infiltration have been implemented individually to reinforce graphene into an Al matrix. To that end, there is also potential to use multiple methods, such as semi-solid stirring and ultrasonic stirring, to achieve graphene reinforcement with excellent properties [16]. For example, Boostani et al. [17,18,19] implemented the combination of semi-solid stirring and non-contact ultrasonic processing to reinforce SiC encapsulated with graphene to enhance the mechanical properties of A356 Al alloy. However, the effect of the processing parameters can be further explored with varying graphene addition, as well as the effect of graphene on alternative aluminum alloys such as A319 Al alloy. As such, the processing of graphene in liquid metals is still in development, especially for varying alloys. Moreover, the current work by the authors implements the utilization of direct contact isothermal ultrasonic stirring with a sonication probe, which may be more suitable for industry applications. Furthermore, it is difficult to isolate the individual strengthening contribution of graphene due to the effect of SiC. Hence, there is potential to explore the individual effects of graphene on the mechanical properties of aluminum alloys.

In the present work, hybrid semi-solid stirring and ultrasonic processing is successfully implemented to reinforce graphene nano-platelets (GNPs) in A319 Al alloy for automotive cylinder heads and engine blocks. To the authors’ knowledge, reinforcement of automotive A319 Al alloy with graphene has not been comprehensively investigated. In contrast to past works, this study demonstrates the isolated effects of semi-solid stirring and direct-contact ultrasonic stirring, as well as the combined effects. Furthermore, the results reveal the individual strengthening contribution of graphene, without the aid of a secondary reinforcement particle. The use of pre-processing techniques, such as ball-milling, were also not required to incorporate graphene in the liquid melt. Most studies involve the use of preprocessing methods such as ball-milling to first manufacture a master-alloy composite prior to casting. This has been found to be successful, although they typically increase the manufacturing time and cost. While there has been significant progress in research involving the incorporation of graphene as a reinforcement particle for Al using liquid metallurgy processes, there is still potential for improvement especially when considering automotive applications as the industry moves towards sustainable electric vehicles.

In this study, a simple and innovative, hybrid semi-solid stirring and ultrasonic processing is implemented to produce an A319/GNP reinforced composite with improved mechanical properties without the need for complex preprocessing methods. A comprehensive analysis on the individual effects of semi-solid stirring and ultrasonic processing on A319 was investigated. Subsequently, the combined effects were then explored and compared to the individual processes. This was achieved using optical microscopy, scanning electron microscopy, X-ray diffraction and tensile tests. The strengthening mechanisms were also studied using existing mathematical models and validated experimentally. The results from this research enable the potential for using cost effective, efficient and simple liquid metallurgy methods to produce aluminum reinforced graphene composites with improved mechanical properties.

## 2. Materials and Methods

Castings of A319-graphene MMCs were produced at the Centre for Near-net-shape Processing of Materials, Toronto, ON, Canada. Graphene particles (graphene nano-platelets) were obtained from XGSciences, Lansing, MI, USA for the production of high-strength GNP reinforced MMCs. The GNPs had an average diameter of 1 µm and thickness of 15 nm, according to the supplier data sheets. In this study, five experimental casting conditions were used and they involved semi-solid processing (SS) or ultrasonic processing (UST). The material designations for the experimental conditions are summarized in Table 1.

For each casting, approximately 1.5 kg of the alloy was melted at 750 °C in a SiC crucible using an electric resistance furnace. Subsequently, a ASTM B108-19 permanent tensile mold was preheated to 300 °C. A combination of semi-solid stirring and ultrasonic stirring was used to incorporate GNPs in the A319 melt. Once the alloy was molten, the furnace was cooled to 590 °C and held at this temperature to allow the A319 alloy to reach semi-solid state. This corresponds to a 0.2 solid fraction for the A319 alloy [20]. Subsequently, the GNPs were preheated to approximately 200 °C to aid in wetting of the particles with liquid Al and remove any moisture [3]. Following this, the GNPs were submerged into the semi-solid alloy and stirred mechanically with an impeller at 1000 RPM for approximately 10 min. Afterwards, the furnace temperature was raised back to 750 °C to allow the alloy to reach molten state. Upon reaching this temperature, ultrasonic treatment was applied directly to the melt using a Sonic Systems (Ilminster, UK) ultrasonic vibration device (model L500) with a frequency of 20 ± 1 kHz. For these experiments the vibrational amplitude was fixed at 24 µm, which was applied to the melt using a titanium alloy sonotrode for approximately 3 min. The ultrasonic treatment was carried out at approximately 750 °C and the sonotrode was immersed in the melt at least 10 mm below the melt surface. Following this, the melt was poured at a temperature of 720 °C into the preheated tensile mold. The chemical composition was determined from the cast samples using an Oxford Instruments Foundry-Master Pro (obtained from NDT Products Ltd., St. Catharines, ON, Canada) optical emission spectrometer and is given in Table 2.

After casting, tensile testing was performed on samples using a Instron United Universal Testing Machine Model STM-50kN (Instron, Massachusetts, United States) according to ASTM B 557M-15. Tensile tests on the dog bone shaped samples (12.95 mm diameter and 50.8 mm gauge length) were carried out with a cross head speed of 12 mm/min at ambient temperature. An extensometer was used to measure strain. After tensile testing, the samples were sectioned for fractography and microstructural analysis. Prior to metallographic preparation, the density of each of the casting conditions was measured using the Archimedes principle on the sectioned samples.

A Nikon Eclipse MA200 (obtained from Buehler, Lake Bluff, IL, United States) metallurgical optical microscope was used to analyze the as-cast grain structure. The samples were prepared for metallography by successive grinding steps using varying levels of SiC papers and subsequently polished using 5 µm alumina, 3 µm diamond suspension, 1 µm diamond suspension and 0.02 µm colloidal silica. The secondary dendrite arm spacing (SDAS) was measured using image analysis with Clemex Vision Pro software (Version Vision PE, Clemex Technologies Inc., Guimond, Longueuil, Quebec, Canada) and the linear intercept method. Furthermore, the GNP particles were examined using a JEOL JSM-6380LV scanning electron microscope (SEM) at 20 keV. Phase microanalysis was carried out using energy dispersive X-ray spectroscopy (EDX). The secondary phases and interfacial reaction products were identified by X-ray diffraction (X’Pert Pro PANalytical X-ray diffractometer). The samples were scanned at 0.05° for 2 s using monochromated Cu K_α_ radiation at 40 mA and 45 kV.

## 3. Results

### 3.1. Microstructural Characterization

#### 3.1.1. As-Cast Microstructure

**Figure 1:**
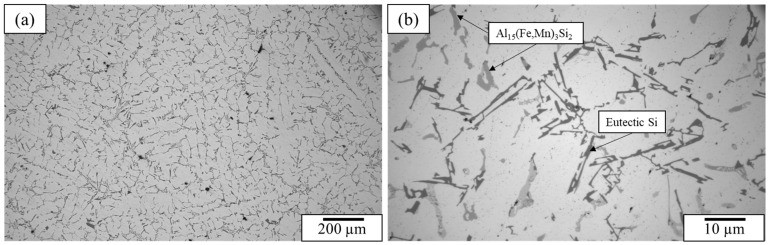
Optical micrographs of the A319 alloy captured at (**a**) 200× and (**b**) 1000× magnification.

Optical microscopy and scanning electron microscopy were used to examine the as-cast grain structure and secondary phases present in the matrix. The grain structure of the castings was found to be dendritic. Furthermore, the as-cast microstructure of the A319 Al alloy consists of α-Al, eutectic Si, θ-Al_2_Cu, and Al_15_(Fe,Mn)_3_Si_2_ phases which were distributed within the interdendritic regions. The optical micrographs of the base A319 alloy can be seen in Figure 1. The phases were also confirmed with XRD as shown in Figure 5. In this study, it was found that the addition of GNPs demonstrated minor changes on the grain structure or the morphology of any secondary phases. The eutectic Si particles remained as coarse acicular flakes and the Al_2_Cu phases were observed in both blocky and lamellar morphologies.

The SDAS decreased slightly with 0.05 wt.% GNP addition (A319 + 0.05 GNP + SS + UST) to 16.0 ± 0.8 µm compared to 19.8 ± 2.4 µm with the base A319 casting (Table 3). This reduction in SDAS demonstrates successful incorporation of GNPs, since the addition of these particles promote grain refinement. Compared to the other conditions, the SDAS remained relatively the same with the highest SDAS reaching 22.2 ± 0.5 µm for the A319 + 0.05 GNP + SS composite. The experiments involving semi-solid stirring alone, resulted in severe particle agglomeration, hence the GNPs were not effectively incorporated in the melt. Similarly, the SDAS was not refined for the A319 + 0.05 GNP + SS and A319 + 0.1 GNP + SS composites demonstrating values of 22.2 ± 0.5 and 20.8 ± 0.6 µm, respectively.

#### 3.1.2. Observation of Graphene Nano-Platelets

**Figure 2:**
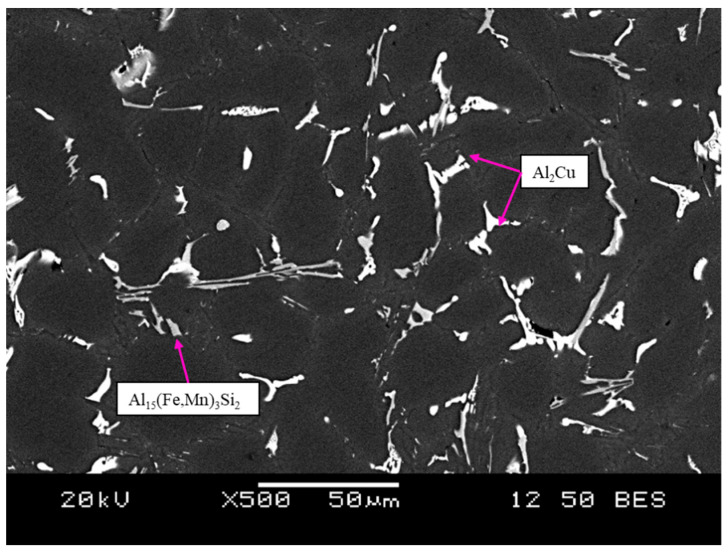
Backscattered SEM image of A319 + 0.05 GNP + SS + UST microstructure at 500×.

Casting experiments were performed to ensure that GNPs were embedded into A319 Al alloy for the production of A319 reinforced GNP composites. For this purpose, SEM analysis was carried out on the samples containing GNPs. Figure 2 demonstrates the general microstructure of the A319 + 0.05 GNP + SS + UST composite. The primary secondary phases observed include Al_2_Cu and Fe-bearing phases. Eutectic Si was also present, as previously shown in Figure 1. However, under the SEM, eutectic Si becomes difficult to observe due to low atomic number. The results show that the Al_2_Cu phases were homogeneously distributed, however they appear to be slightly fragmented as micro-spherical forms of Al_2_Cu are present near the larger blocky phases. This is may be a result of the sonication effect, which is known to refine secondary phases due to cavitation induced by high frequency vibration.

**Figure 3:**
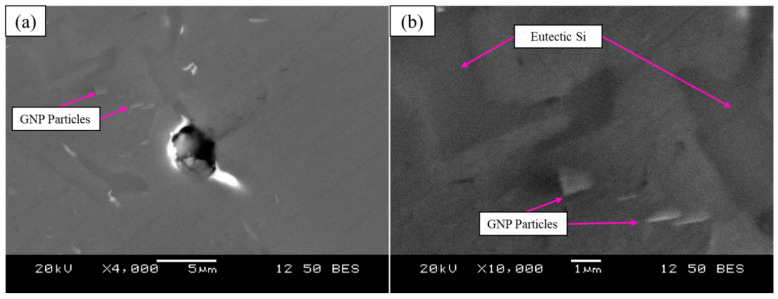
Backscattered SEM images of A319 + 0.05 GNP + SS + USTcomposite showing graphene nano-platelets embedded in Al matrix near eutectic Si at (**a**) 4000× and (**b**) 10,000× magnification.

**Figure 4:**
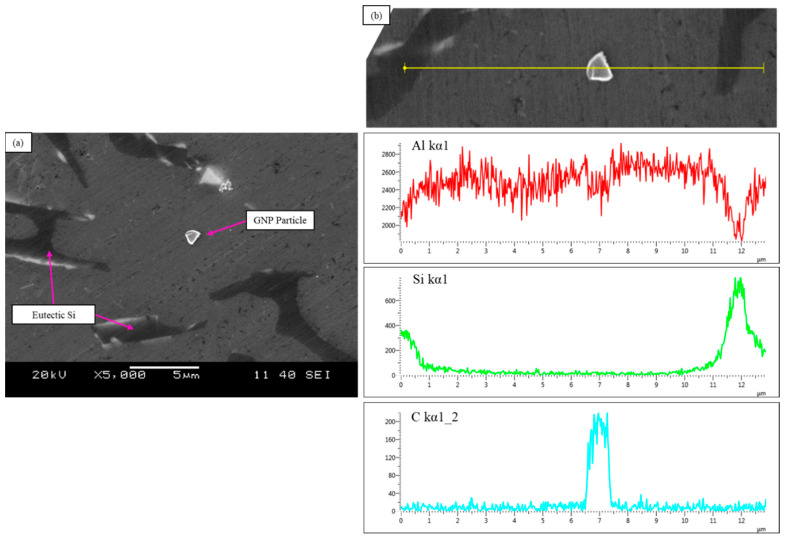
(**a**) Secondary electron image of GNP particle and (**b**) corresponding EDX linescan for A319 + 0.05 GNP + SS + UST composite.

**Figure 5:**
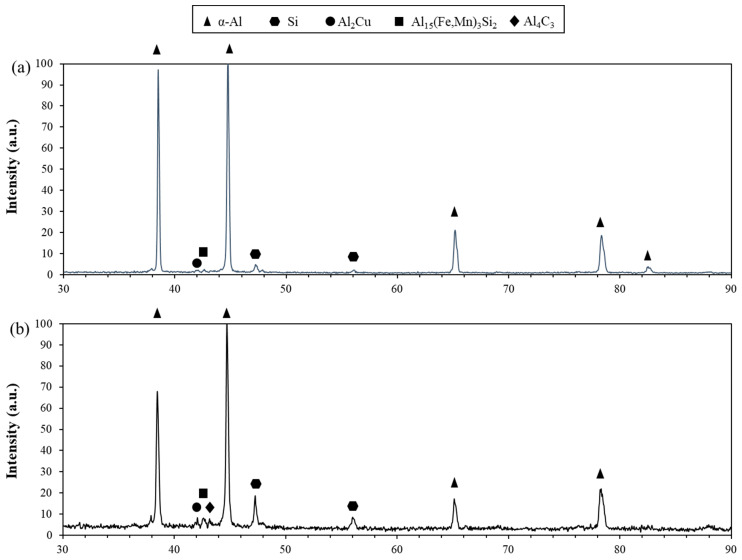
XRD analysis of (**a**) A319 base alloy and (**b**) A319 + 0.05 GNP + SS + UST composite.

Additionally, GNP particles were observed in the matrix of the A319 Al alloy using SEM as seen in Figure 3. The GNPs were embedded longitudinal to the matrix examined under the SEM. Although, a number of particles appeared to be embedded at an angle to the surface (Figure 3b), potentially indicating a different plane. Furthermore, the analysis revealed that the GNP particles were typically observed near a eutectic Si phase as seen in Figure 4 and Figure 5. Since, A319 predominantly consists of eutectic Si this may be related to the solidification mechanism whereby the GNPs are pushed into the interdendritic regions. Energy dispersive X-ray spectroscopy (EDX) was also performed to confirm the presence of GNP particles. This can be seen in Figure 4, where a different region showing the presence of GNPs is presented with a corresponding EDX linescan of a GNP particle (Figure 4b). The EDX linescan initially demonstrates an increase in energy corresponding to a eutectic Si particle with increasing distance, which is then followed by a peak in C energy that follows the contour of the GNP particle (platelet morphology).

The addition of GNPs can also promote the formation of interfacial products such as Al_4_C_3_. The high temperature processing, coupled with a relatively long processing time promotes breaking down of C atoms from graphene. Hence, it is thermodynamically favorable that a reaction can occur between C and available Al in the melt to produce Al_4_C_3_. X-ray diffraction was implemented to confirm the presence of Al_4_C_3_ with the addition of GNPs (Figure 5), since it is known that Al_4_C_3_ can deteriorate the mechanical properties of aluminum matrix composites. This was observed in a sample from the A319 + 0.05G + SS + UST condition, where a peak was identified at 43° (Figure 5b). Compared to the base A319 casting, this peak does not occur at the same angle as seen in Figure 5a. Previous research has reported Al_4_C_3_ formation with diffraction angles of 31, 32, 33, 40 and 43° [21]. Similarly, this also correlates well to the findings by Alipour et al. [12] where the authors observed a peak at 43° corresponding to GNP addition, although it was not explicitly identified as Al_4_C_3_. However, identification of Al_4_C_3_ is often difficult even with the use of XRD. In this case, Al_4_C_3_ was occasionally observed in limited quantity according to the XRD results. This was likely due to the very low addition of GNP particles. Additionally, localized inhomogeneity could be a potential cause of the observation of Al_4_C_3_ in very limited quantity. Of note, it should be mentioned that the corresponding Al_4_C_3_ peak (43°) closely overlaps with the Al_15_(Fe,Mn)_3_Si_2_ secondary phase, while it is possible for the Al_4_C_3_ interfacial product to reduce the mechanical properties, this was not observed in this study.

### 3.2. Mechanical Properties

**Figure 6:**
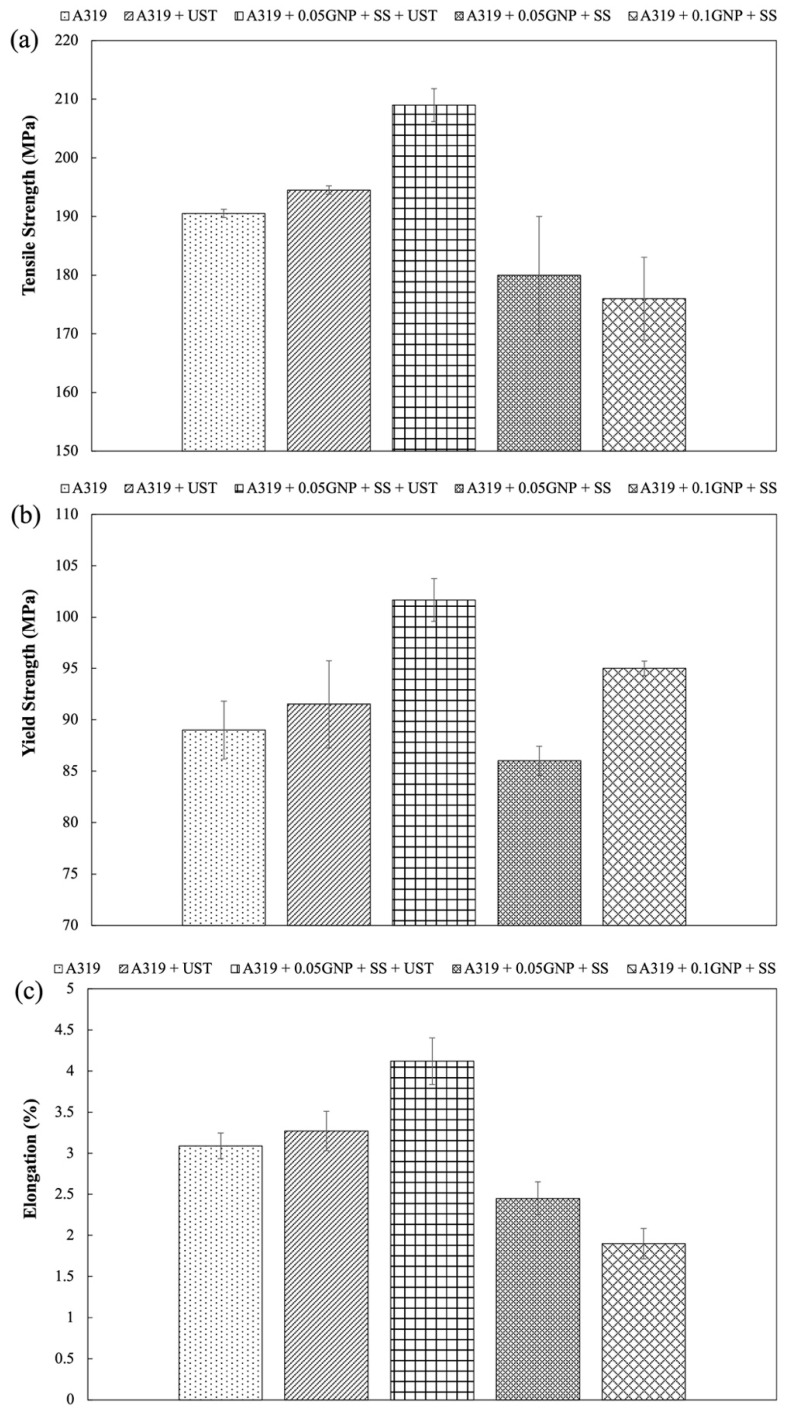
(**a**) Ultimate tensile strength, (**b**) yield strength and (**c**) elongation of cast samples.

In this study, tensile testing was carried out to determine the mechanical properties of the composite castings. The mechanical properties of the casting conditions are shown in Figure 6. The base A319 condition exhibited a UTS of approximately 190 MPa. A319 with ultrasonic processing was also carried out to isolate the effects of ultrasonic processing on the microstructure and mechanical properties. It was found that there was a minor increase in UTS to 195 MPa for the A319 + UST samples. This can be attributed to the degassing effect and inclusion removal of ultrasonic processing, thereby promoting a cleaner melt through the removal of hydrogen gas and oxides. Experiments were also carried out to demonstrate the effect of semi-solid processing on incorporating GNPs in the melt. This was done for additions of 0.05 wt.% and 0.1 wt.% GNPs. The main purpose of using semi-solid processing was to prevent GNP particles from floating to the melt surface. This was found to be an effective method in preventing particle floating, however the results demonstrated a reduction in UTS to 180 and 176 MPa for the A319 + 0.05 GNP + SS and A319 + 0.1 GNP + SS samples, respectively. Conversely, combining both ultrasonic processing and semi-solid processing resulted in an increase in UTS for an addition of 0.05 wt.% GNPs. The A319 + 0.05 GNP + SS + UST samples resulted in a UTS of 209 MPa. This was attributed to the combined effects of semi-solid processing in preventing particle floating and the ability of ultrasonic processing to de-agglomerate and distribute GNPs in the melt.

In addition to the findings on the UTS, the yield strength was also the highest for the A319 + 0.05 GNP + SS + UST samples. The YS for these samples was measured at approximately 119 MPa compared to the base A319 cast samples, which demonstrated a YS of 107 MPa. As shown in Figure 6b, the other conditions resulted in similar YS values to the base condition and did not show improvements. The ductility of the conditions also followed a similar trend to the UTS results, displayed in Figure 6c. The ductility of the base A319 condition was measured at 3.1%. Additionally, the A319 + UST samples exhibited a slight increase in elongation at 3.3%, likely due to its degassing effect. While degassing is likely taking place during melt treatment, ultrasonic processing was not found to refine the grains as previously discussed. However, for the conditions involving semi-solid processing individually produced samples with reduced ductility compared to the base A319 casting. With semi-solid processing the A319-0.05 GNP + SS and A319-0.1 GNP + SS, the ductility reduced to approximately 2.4 and 1.9%, respectively, indicating a brittle fracture behavior. In contrast, the results showed an increase in ductility to 4.1% for the A319 + 0.05 GNP + SS + UST composite using the combination of semi-solid and ultrasonic processing.

The results suggest that while semi-solid processing can prevent floating of GNP particles to the melt surface, the process is not effective in de-agglomerating the GNP particles. Hence, ultrasonic processing is required de-agglomerate and distribute GNPs in the melt. As demonstrated by the UTS and YS results, the combination of ultrasonic processing and semi-solid processing demonstrates potential for producing AMCs reinforced with graphene with improved mechanical properties. In summary, the tensile test results demonstrated 10, 11 and 32% improvements in UTS, YS, and ductility of A319 reinforced with 0.05 wt.% addition of graphene through using hybrid semi-solid and ultrasonic processing.

### 3.3. Strengthening Models

In this study, theoretical models were used to determine the mechanisms for enhancing YS of the A319 + 0.05 GNP + SS + UST composite. A modified shear-lag and enhanced dislocation density model, developed by Fadavi Boostani et al. [18] was used to accurately predict the YS and the subsequent strengthening contributions as seen in Equation (1). It should be noted that due to the size of the particles used in the study, that the Orowan strengthening component was neglected.

(1) $$\sigma_{Y}^{Gr}=\sigma_{Y}^{Al}\left(1+\omega_{L}\right)\left(1+\omega_{T}\right)+\sigma_{Y}^{Hall-Petch}\;$$

Equation (2) is composed of the strengthening effects in the form of load bearing ($\omega_{L}$), thermally induced dislocations ($\omega_{T}$), and Hall-Petch mechanisms on the yield strength of the A319 alloy used in this study to approximate the yield strength of A319 + 0.05 GNP + SS + UST. The Hall-Petch strengthening was approximated using Equation (2).

(2) $$\sigma_{Y}^{Hall-Petch}=\sigma_{i}+kD^{-\frac{1}{2}}\;$$

where $\sigma_{i}$ and k are the intrinsic stress ($\sigma_{i}$ = 15.7 MPa) and material constant (k = 0.068 MPa/M^0.5^) [22]. Using the measured SDAS in Table 3 for A319 + 0.05 GNP + SS + UST (D = 16 µm), the strengthening contribution from the Hall-Petch relationship exhibits a 16.2 MPa enhancement in YS. Of note, SDAS was measured for the samples in this study which has been found to accurately follow the Hall-Petch relationship in Al-Si cast alloys according to Ghasemalli et al. [23].

The load transfer from the Al matrix to graphene particles is known to contribute to the strengthening of the matrix material, this phenomenon can be expressed by Equation (3). This model is modified to account for the platelet-like morphology of graphene sheets or graphene nano-platelets. Hence, the load bearing parameter is defined as:

(3) $$\omega_{L}=\sqrt{\frac{2G_{m}L^{2}}{E_{f}\lambda_{eff}t}}\;$$

where $G_{m}$, $E_{f}$, L, and t are the matrix shear modulus, Young’s modulus, particle length and particle thickness, respectively. For the load bearing phenomenon, it is also important to take into account the inter-particle spacing, which is defined here as $\lambda_{eff}$:

(4) $$\lambda_{eff}=0.931\sqrt{\frac{0.306\pi dt}{V_{Gr}}}-\frac{\pi d}{8}-1.061t\;$$

where d, t, and $V_{Gr}$ are the particle length, thickness, and volume fraction, respectively. Using the values d = 1 µm, t = 15 nm, and $V_{Gr}$ = 0.00063 results in an inter-particle spacing $\lambda_{eff}$ = 4.59 µm. Then, using the calculated theoretical inter-particle spacing ($\lambda_{eff}$) value and inserting into Equation (3), $\omega_{L}$ = 0.03.

There is a substantial difference in the coefficient of thermal expansion (CTE) of graphene and aluminum. This leads to an increase in dislocation density as a result of a plastic strain developed from the large ΔCTE. This can be expressed by Equation (5):

(5) $$\omega_{T}=\frac{1.25G_{m}b}{\sigma_{Y}^{Al}}\sqrt{\frac{12\left(T_{Fabrication}-T_{Test}\right)\left(\alpha_{m}-\alpha_{Gr}\right)V_{Gr}}{bd\left(1-V_{Gr}\right)}}\;$$

where $G_{m}$, b, d and $\sigma_{Y}^{Al}$ are the matrix shear-modulus, the burgers vector, the diameter of graphene particles and the yield strength of the unreinforced A319 alloy, respectively. $T_{Fabrication}$ is the processing temperature (750 °C), while $T_{Test}$ is the test temperature (300 °C). Using the values from Table 4, $\omega_{T}$ = 0.045.

Inserting the values $\omega_{L}$ = 0.03, $\omega_{T}$ = 0.045 and the YS enhancement from the Hall-Petch relationship into Equation (1) results in a total theoretical YS of 130 MPa. This is indeed close to the experimental YS value of 119 MPa for the A319 + 0.05 GNP + SS + UST composite, as previously discussed. This is approximately an 8.5% deviation from the theoretical YS determined from the model discussed. Hence, the mathematical models can accurately predict YS of AMCs reinforced with graphene.

### 3.4. Fracture Behavior

**Figure 7:**
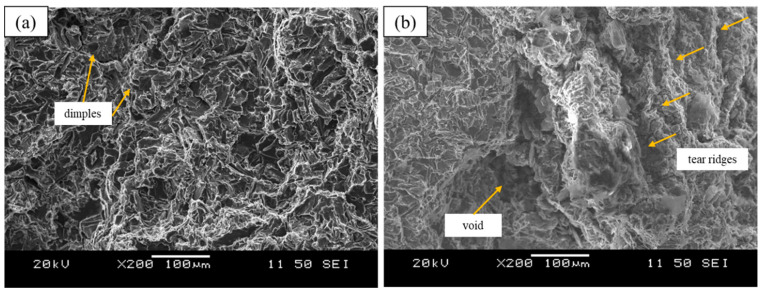
Fracture surface of (**a**) A319-0.05 GNP + SS + UST and (**b**) A319-0.1 GNP + SS samples.

Fractography using SEM was carried out after tensile testing the cast samples. Figure 7a,b present the fractographic images of the A319-0.05 GNP + SS + UST and the A319-0.1 GNP + SS samples, identified as the best and worst conditions in this study. The A319-0.05 GNP + SS + UST samples exhibited a UTS, YS, and ductility of 209 MPa, 119 MPa, and 4.1%, respectively, as previously discussed. As a result, the fracture surface of these samples exhibited a ductile behavior compared to the other conditions as demonstrated by the dimples across surface (Figure 7a). This suggests that the GNPs displayed good interfacial bonding at the Al interface. Hence, this also contributed to the ductile fracture behavior as de-lamination of GNPs or micro-cracks were not observed. Fine cleavage planes can also be observed across the sample fracture surface. The A319-0.1 GNP + SS samples displayed UTS, YS, and ductility values of 176 MPa, 111 MPa, and 1.9%, respectively. The results show that the fracture surface exhibits a brittle fracture, presented in Figure 7b. This was due to agglomeration of GNP particles which resulted in a large void, which was a site for crack initiation and propagation. Additionally, tear ridges can be observed on the fracture surface which also show the direction of the crack propagation, whereby the fracture propagates away from the void region where the GNPs agglomerated. The results suggest that semi-solid processing should be accompanied by subsequent ultrasonic processing when adding the GNP particles. The semi-solid processing inhibits floating of particles to the melt surface, however during this process the particles have a tendency to agglomerate. Hence, following up this process with ultrasonic processing enables de-agglomeration of the GNP particles as shown in Figure 7.

## 4. Discussion

This study explores a processing route using a liquid metallurgy method to produce a A319 graphene reinforced MMC. The purpose of this study is to determine a process to effectively insert GNPs in automotive A319 Al alloy through liquid metallurgy, specifically casting. The results demonstrate that ultrasonic processing and semi-solid processing individually cannot effectively distribute GNPs in the liquid melt. Hence, a combination of both ultrasonic processing and semi-solid processing was performed to achieve effective addition of GNP particles. In order to demonstrate the effectiveness of combining both methods, casting conditions that isolate each process and the base condition were carried out. As a result, an A319-0.05 wt.% GNP composite (A319 + 0.05 GNP + SS + UST) was successfully produced with improved mechanical properties. This was achieved via a simple and innovative hybrid semi-solid and ultrasonic processing method. In order to demonstrate the synergistic effect of the hybrid method, castings of base A319, A319 with UST, and A319 with SS processing were also produced. Hence, these conditions demonstrate the isolated effects of UST and SS while also comparing to the A319 alloy without additions.

### 4.1. Effect of Processing and GNP Addition on A319 As-Cast Microstructure

The SDAS measurements of A319, A319 + UST, A319 + 0.05 GNP + SS, and A319 + 0.1 GNP + SS were measured as 19.8 ± 2.4, 21.8 ± 2.0, 22.2 ± 0.5, and 20.8 ± 0.6 µm, respectively (Table 3). Between these conditions the SDAS remained relatively unchanged. This indicated that UST processing alone did not refine the A319 alloy. Furthermore, the castings involving SS processing and GNP additions also did not show refinement in SDAS. While it is expected that GNP additions promote refinement of the alloy microstructure, this may be inhibited by agglomeration of GNPs. Agglomeration was indeed present in the A319 + 0.05 GNP + SS and A319 + 0.1 GNP + SS as demonstrated by the reduced mechanical properties (Figure 6) and the fracture surface (Figure 7b). In contrast, hybrid SS and UST processing enabled effective and homogeneous addition of GNPs into the melt. The SDAS of A319 + 0.05 GNP + SS + UST was measured as 16.0 ± 0.8 µm, which exhibit a slight reduction compared to the base A319 alloy. It has been previously reported that graphene, due to its high thermal conductivity, can change the solidification mechanism. As a result, it is possible for the particles to affect the temperature gradient ahead of the solidification front potentially leading to reduced grain size [18,25,26], while the SDAS refinement is minor, it still demonstrates slight refinement compared to the other conditions and it should be noted that only 0.05 wt.% of GNPs was added.

SEM analysis was carried out for observation of GNPs and analysis of the GNP interface with the Al matrix. The results showed that GNP particles were embedded in the Al matrix, typically observed near eutectic Si particles. This may indicate that during solidification graphene particles were pushed towards the interdendritic regions, as opposed to pinning at the boundaries of the dendrites. As previously discussed, this suggests that it is related to solidification mechanisms which are affected by the high thermal conductivity of graphene. Additionally, SEM and EDX confirmed the platelet morphology of the observed particle as well as the chemistry, demonstrated in Figure 4b. Figure 3b and Figure 4a also demonstrates that the GNP particles have a strong and cohesive interface with the Al matrix. As a result, there was no observation of delamination between at the Al-GNP interface. This also correlates well to the fracture behavior of A319 + 0.05 GNP + SS + UST, which exhibited a relatively ductile fracture without the observation of micro-cracks, shown in Figure 7a.

### 4.2. Analysis of Interfacial Reactions

With the addition of reinforcing particles such as GNPs, it is also important to consider the interfacial interaction between the reinforcement particle and the matrix. The addition of reinforcement particles at high processing temperatures may produce reaction products, as the thermodynamic stability becomes weaker with elevated temperatures. Namely, Al_4_C_3_ can form if the GNP particles break down during the processing. This also is more likely to occur with increased processing times. Because preparation of the A319 + 0.05 GNP + SS + UST composite involves a multi-stage melt treatment, the processing time was longer compared to a conventional casting process. Figure 5 shows the XRD results of the A319 + 0.05 GNP + SS + UST composite, showing the peaks and corresponding reaction products. The peaks identified are standard for α-Al, Al_2_Cu, Si and Al_15_(Fe,Mn)_3_Si_2_. However, the results demonstrated that at 43° there was a peak, likely corresponding to Al_4_C_3_. Despite this, it was difficult to identify using XRD and the corresponding database likely due to the low addition of GNPs. The results correlate well to a study by Lee et al. [21] on the formation of Al_4_C_3_ in Al-Si alloys, where the authors determined that the transition temperature for the formation of Al_4_C_3_ is approximately 620 °C. In this study, the processing temperature reached a maximum of 750 °C, hence it is thermodynamically favorable for Al_4_C_3_ to form during the high temperature processing. Of note, it is likely that the amount of Al_4_C_3_ is minimal due to the low addition of 0.05 wt.% GNPs. Moreover, localized inhomogeneity and variation in composition, likely resulted in the interesting observation of Al_4_C_3_ in limited quantity. The direct observation of Al_4_C_3_ will be carried out using high-resolution transmission electron microscopy (HR-TEM) as part of a separate on-going study by the authors. Lastly, the limited presence of Al_4_C_3_ was not found to deteriorate the mechanical properties of the A319 + 0.05 GNP + SS + UST composite.

### 4.3. Enhancement of Mechanical Properties

In this study, the mechanical properties were evaluated for each of the castings (Figure 6). The results showed enhanced mechanical properties for the samples undergoing hybrid semi-solid and ultrasonic processing (A319 + 0.05 GNP + SS + UST). The UTS and YS of A319 + 0.05 GNP + SS + UST displayed a 10 and 11% increase, respectively, compared to the base A319 casting. A considerable increase in strength for a low addition of GNPs. Additionally, the ductility also increased from 3.1% to 4.1% compared to base A319, which is a 32% increase. The increase in mechanical properties demonstrate the effectiveness of the GNP particles as a reinforcement material. The results suggest that the GNPs were effectively incorporated in A319 Al alloy using the hybrid method. This was shown in the SEM results whereby the GNP particles were observed and embedded in the Al matrix. The GNPs were typically oriented longitudinal to the analyzed surface; however, GNPs were also observed oriented at an angle to the surface (Figure 3b). Additionally, the GNPs displayed good interfacial bonding at the Al interface which correlates well to the mechanical properties. The interface between the GNP particle and the Al matrix was well observed in Figure 4. This also contributed to the ductile fracture behavior as de-lamination of GNPs or micro-cracks were not observed in this study. Additionally, clustering of GNPs was not observed under the SEM, which indicates that ultrasonic processing was effective in de-agglomerating clusters of GNPs into individual particles. The efficiency of the strengthening by GNP reinforcement likely depends on which planes the GNPs will inhabit as the properties are more dominant when the load is applied in the transverse direction. Modeling and prediction of the orientation of GNPs and solidification mechanisms should be further explored as this helps to understand how to fully optimize the mechanical properties.

As previously discussed, GNP particles were observed under the SEM embedded in the Al matrix. While GNPs are a contributor to the strength enhancement of A319, it should be noted that the sonication process also degasses the melt. Hence, there is an additional benefit of ultrasonic processing when producing a composite through casting. Interestingly, ultrasonic processing alone was found to exhibit slight improvements in the mechanical properties of A319, where the UTS and YS slightly increased from 190 to 195 MPa and 107 to 109 MPa, respectively. As previously mentioned, the slight improvement in mechanical properties is due to degassing of the melt. Although, it was found that UST processing did not refine the A319 alloy, which has been reported to occur in other alloys such as Mg [27,28].

In order to isolate the effects of semi-solid processing, castings involving the addition of GNPs were carried out only using semi-solid processing as the melt treatment. For both the A319 + 0.05 GNP + SS and A319 + 0.1 GNP + SS composites, the UTS decreased by 5.3 and 7.4%, respectively. Similarly, the fracture behavior was brittle in both castings with the worst condition (A319 + 0.1 GNP + SS) having a ductility of 1.9%. This was a result of severe agglomeration of the GNP particles, which can be seen in Figure 7b. In this study, semi-solid processing was used to prevent floating of GNP particles due to their much lower density compared to Al. While this process was effective in doing so, it is evident that mechanical mixing during semi-solid processing is not effective in preventing agglomeration of GNP particles. Hence, it is important to follow this process with ultrasonic processing to de-agglomerate and distribute the particles.

The strengthening contributions were also analyzed using theoretical mathematical models. The main strengthening contributors to YS are Hall-Petch, load bearing mechanism, and thermally induced dislocations as a result of CTE mismatch between graphene particles and Al. The theoretical YS enhancement from each mechanism was calculated to be 16.2, 3.0 and 4.8 MPa, respectively. Hence, the highest contribution to strength was due to the dendritic grain refinement through the Hall-Petch relationship. This is followed by CTE mismatch between graphene and the Al matrix, then by load bearing mechanism. As a result, the total contribution to YS enhancement is 24 MPa resulting in a theoretical YS of approximately 130 MPa. Although, compared to the experimental result there is a deviation by approximately 8.5%. Hence, it can be suggested that the model can be considered to predict the YS of AMCs reinforced with GNPs.

The discrepancy in the YS between the theoretical calculation and the experimental result can be attributed to some factors. The total calculated volume fraction does not fully represent that of the experiment due to losses throughout the casting process. The orientation of GNPs is not accounted for in the model, for example the strength properties are more dominant in the transverse loading direction compared to parallel with the corresponding plane. Furthermore, it is not fully known which planes the GNPs will inhabit during solidification, although it is expected that to achieve optimal strengthening enhancement this should be the (111) aluminum slip plane. The formation of interfacial products, such as Al_4_C_3,_ is also not accounted for in the theoretical models. It can be expected that such compounds can act as either a strengthening phase or deteriorate the YS.

The results of this study demonstrated the effectiveness of reinforcing A319 with GNPs through a simple liquid metallurgy technique. To the authors’ knowledge, reinforcement of automotive A319 Al alloy with graphene has not been comprehensively investigated. As a result, the findings in this study further contributes to light weight and development of high-strength materials for automotive powertrain components. The mechanical properties of A319 were enhanced with cost effective and low additions of GNPs using a hybrid semi-solid and ultrasonic melt treatment during the casting process. As a result, the formation of Al_4_C_3_ was minimal with the addition of 0.05 wt.% GNPs. This was an important finding due to the negative effect of Al_4_C_3_ on the mechanical properties. Lastly, it was found that theoretical strengthening models resulted in values close to the experimental results, although adjustments should be made to account for the orientation of GNPs in the metal matrix.

This study is significant since it clearly demonstrated that high cost and time-consuming preprocessing methods such as ball milling, spark plasma sintering and other methods are not required for efficient production of GNP reinforced Al composites. This is especially important for developing high strength materials for the automotive industry. Therefore, cost effective and efficient cast Al-GNP composites prepared through liquid metallurgy techniques, such as casting, can be used for next generation automotive vehicles leading to light weight and increased performance.

## 5. Conclusions

This research investigated a processing route using a casting process to produce a A319 graphene reinforced MMC. As a result, an A319-0.05 wt.% GNP composite (A319 + 0.05 GNP + SS + UST) was successfully cast with improved mechanical properties. This was achieved via a hybrid semi-solid and ultrasonic treatment method. In order to demonstrate the synergistic effect of the hybrid method, castings of base A319, A319 with UST, and A319 with SS processing were also produced. The alloy microstructure, analysis of graphene in the Al matrix, and the mechanical properties of the castings were examined. Subsequently, the strengthening contributions were also analyzed using theoretical mathematical models, which were then validated with the experimental results. The results from this research enable further understanding of a simple liquid metal processing route to produce aluminum matrix composites reinforced with graphene for high performance next generation automotive vehicles. The following conclusions can be drawn from this study:

1. The combination of semi-solid processing and ultrasonic processing was an effective and efficient method for the incorporation of GNPs in A319 Al alloy. This was attributed to prevention of particle floating during semi-solid stirring and the ability of ultrasonic processing to effectively de-agglomerate and distribute the GNPs in the liquid melt.
2. Semi-solid processing of A319 Al enabled the prevention of floating of GNPs to the melt surface, however it was ineffective in de-agglomerating GNP particles. This resulted in excessive agglomeration of GNPs promoting a brittle fracture and a reduction in mechanical properties.
3. Addition of 0.05 wt.% GNPs to A319 Al using the hybrid semi-solid and ultrasonic processing method resulted in an enhancement in UTS, YS, and ductility by 10, 11 and 32%, respectively. The SEM results revealed good interfacial bonding between GNP and Al-matrix. This was an important finding due to the very low addition of GNPs, which suggests a cost-effective incorporation of particles accompanied by the enhancement in mechanical properties.
4. X-ray diffraction results indicate the limited presence of Al_4_C_3_ with the addition of GNPs, which did not lead to any reduction in mechanical properties in the A319 + 0.05 GNP + SS + UST composite. This was attributed to localized inhomogeneity and variation in composition, likely resulted in the interesting observation of Al_4_C_3_ in limited quantity.
5. The strengthening contributions for the A319 alloy reinforced with 0.05 wt.% GNPs (A319 + 0.05 GNP + SS + UST) were evaluated using mathematical models. The strength enhancements were primarily attributed to the Hall-Petch effect, followed by CTE mismatch and load bearing mechanism.
6. The mathematical models used to estimate the YS of the alloy corresponded well to the experimental results. The theoretical YS was calculated as 130 MPa, whereas the experimental YS was measured as 119 MPa. Thus, there is an 8.5% deviation and it can be suggested that the model is accurate in predicting YS of aluminum matrix composites reinforced with GNPs.

## Figures and Tables

**Table 1:** Summary of casting conditions and designation.

Condition	GNP Addition (wt.%)	Semi-Solid (SS)	Ultrasonic Processing (UST)
A319	-	-	-
A319 + UST	-	-	*
A319 + 0.05 GNP + SS + UST	0.05	*	*
A319 + 0.05 GNP + SS	0.05	*	-
A319 + 0.1 GNP + SS	0.1	*	-

* indicates that the process was applied.

**Table 2:** Average chemical composition of A319 alloy used in this study.

Element	Si	Cu	Mg	Ti	Fe	Mn	Zn	Ni	Al
wt.%	6.21	2.87	0.04	0.11	0.58	0.34	0.61	0.18	Bal.

**Table 3:** Summary of average SDAS measurements for casting conditions.

Sample	SDAS (µm)
A319	19.8 ± 2.4
A319 + UST	21.8 ± 2.0
A319 + 0.05 GNP + SS + UST	16.0 ± 0.8
A319 + 0.05 GNP + SS	22.2 ± 0.5
A319 + 0.1 GNP + SS	20.8 ± 0.6

**Table 4:** Parameters used for calculating strengthening contributions [4,22,24].

Parameter	Unit	Al	Graphene
Burgers vector (b)	nm	0.25	-
Thermal expansion coefficient (α)	×10^−6^/°C	21.4	>−6
Shear modulus (G)	MPa	25	250
Young’s modulus	GPa	70	1000
Intrinsic stress ($\sigma_{i}$)	MPa	15.7	-
Material constant (k)	MPa/M^0.5^	0.068	-

## Data Availability

Not applicable.
